# Rose bush leaf and internode expansion dynamics: analysis and development of a model capturing interplant variability

**DOI:** 10.3389/fpls.2013.00418

**Published:** 2013-10-24

**Authors:** Sabine Demotes-Mainard, Jessica Bertheloot, Rachid Boumaza, Lydie Huché-Thélier, Gaëlle Guéritaine, Vincent Guérin, Bruno Andrieu

**Affiliations:** ^1^Institut National de la Recherche Agronomique, UMR1345 IRHSBeaucouzé, France; ^2^Agrocampus-Ouest, UMR1345 IRHSAngers, France; ^3^Université d'Angers, UMR1345 IRHSAngers, France; ^4^SFR4207 QUASAVAngers, France; ^5^Institut National de la Recherche Agronomique, UMR1091 EGCThiverval-Grignon, France; ^6^AgroParisTech, UMR1091 EGCThiverval-Grignon, France

**Keywords:** *Rosa hybrida* L., individual plant, phytomer, model, elongation kinetics, leaflet size, internode length, growth

## Abstract

Rose bush architecture, among other factors, such as plant health, determines plant visual quality. The commercial product is the individual plant and interplant variability may be high within a crop. Thus, both mean plant architecture and interplant variability should be studied. Expansion is an important feature of architecture, but it has been little studied at the level of individual organs in rose bushes. We investigated the expansion kinetics of primary shoot organs, to develop a model reproducing the organ expansion of real crops from non-destructive input variables. We took interplant variability in expansion kinetics and the model's ability to simulate this variability into account. Changes in leaflet and internode dimensions over thermal time were recorded for primary shoot expansion, on 83 plants from three crops grown in different climatic conditions and densities. An empirical model was developed, to reproduce organ expansion kinetics for individual plants of a real crop of rose bush primary shoots. Leaflet or internode length was simulated as a logistic function of thermal time. The model was evaluated by cross-validation. We found that differences in leaflet or internode expansion kinetics between phytomer positions and between plants at a given phytomer position were due mostly to large differences in time of organ expansion and expansion rate, rather than differences in expansion duration. Thus, in the model, the parameters linked to expansion duration were predicted by values common to all plants, whereas variability in final size and organ expansion time was captured by input data. The model accurately simulated leaflet and internode expansion for individual plants (RMSEP = 7.3 and 10.2% of final length, respectively). Thus, this study defines the measurements required to simulate expansion and provides the first model simulating organ expansion in rosebush to capture interplant variability.

## Introduction

Plant architecture constitutes the interface by which the plant gathers resources and perceives signals from its environment, which in turn modify plant architecture. In ornamental crops, such as rose bush, plant architecture is important in its own right, because it conditions plant visual quality, largely accounting for consumer choice (Boumaza et al., 2010). The management of plant architecture, by manipulation of the environment, in particular, is therefore an important issue for rose bush growers. The development of plant architecture should be studied at two levels: that of the crop, the level at which management operates and interactions occur between neighbors, and the individual plant level, as it is individual plants that are sold. A knowledge of mean values for a crop is therefore not sufficient; the variability between plants within a crop should also be characterized. To investigate the relationships between rose bush architecture and its environment, a functional-structural plant model (FSPM) of rose bush would be a powerful tool. Firstly, FSPMs account for plant architecture at the individual plant level; secondly, when coupled with phylloclimate models, FSPMs enable to estimate the physical environment actually perceived by aerial organs, which is heterogeneous within and between plants (Chelle, 2005).

Plant architecture firstly depends on bud fate, which determines the number and location of shoots; furthermore on the initiation, expansion and (re)orientation of internodes and leaves, and on floral transition, which determines the time of flowering and the number of flowers. All these traits have a potential impact on the visual quality of rose bush. The architectural traits that we wished to model in this study relate to the expansion of the main organs of the aerial vegetative apparatus: the stems and leaves. In addition to their physiological functions, the stems determine the shape of the plant (top-sided shape, symmetry). Together with the leaves, they determine the compactness of the plant. Both these traits are thus important elements of visual quality (Boumaza et al., 2009). The expansion of an individual organ is characterized by the time of organ expansion, the expansion rate and expansion duration. Within a shoot, these traits follow gradients according to organ position which need to be characterized.

Expansion features vary between species, and specific studies are therefore required. The kinetics of leaf and internode expansion at the phytomer level have never been described for rose. Several models describing architectural variables have been developed for cut-flower roses. A first group of models predicts variables at the level of the whole shoot, such as total stem length and basal stem diameter (Hopper et al., 1994; Costa and Heuvelink, 2003; Oki et al., 2006) or morphological quality classes (Morisot, 1996). Structural descriptions have been refined further in more mechanistic models (Lieth and Pasian, 1991; Dayan et al., 2004), mostly with a view to predicting the harvest of rose flowers more accurately. Considerable effort has thus gone into modeling photosynthesis and assimilate partitioning, but the kinetics of expansion of individual organs and its variation with respect to organ position have not been described. The FSPM of Buck-Sorlin et al. (2011) for cut-flower roses included the expansion kinetics of individual organs, but the paper focuses on simulating the local light climate and photosynthesis and does not present results for organ expansion. In rose bush, Demotes-Mainard et al. (2009) have described the coordination of leaflet and internode expansion kinetics as a function of position along the primary shoot. However, these preliminary results were obtained for only one set of growth conditions and relate exclusively to the behavior of an average 11-phytomer plant. With a view to developing a FSPM for rose bush one step consists in modeling organ expansion. Therefore, additional knowledge of the kinetics of individual organs must be obtained.

As in many species, organ size in rose is influenced by environmental factors, such as water (Demotes-Mainard et al., 2013), or nitrogen (Ashok and Rengasamy, 2000; Huché-Thélier et al., 2011) availability, light quality (Rajapakse and Kelly, 1994; Maas and Bakx, 1995) and intensity (Hopper and Hammer, 1991; Bredmose, 1993; Maas and Bakx, 1995), mechanical stimulation (Morel et al., 2012), and genotype (Morel et al., 2009). Current knowledge of the effects of environmental factors is not sufficient to predict internode or leaf size in a range of environments. However, one way to reconstruct the plant architecture of experimental crops accurately for the investigation of plant functioning involves using a model that simulates architecture with data obtained from experimental crops. These experimental data must capture the variability induced by the environment and not predicted by the model. This approach has been applied successfully in several studies [for example in Baccar et al. (2011), to study the effect of wheat architecture on *Septoria tritici* epidemics; Kahlen and Stützel (2011), to study the photo-modulation of cucumber internode elongation]. The input data used to reconstruct architecture are generally the means obtained for plant samples. We suggest that the use of data from individual plants should make it possible to explain and reproduce not only the mean plant characteristics, but also the interplant variability. Using individual data would not increase the time required for data acquisition, because architectural data are necessarily measured at the individual plant and organ level. For the correct reproduction of individual plants, the input data should capture interplant variability. This requires good knowledge of variations of the expansion kinetics of individual organs between plants.

The aim of this work was (i) to investigate the expansion kinetics of rose bush primary shoot internodes and leaves and its variation between phytomer positions and between plants and (ii) to propose an empirical model reproducing organ expansion kinetics that accounts for interplant variability and makes use of non-destructive, easy-to-measure input variables.

## Materials and methods

### Plant material and growing conditions

*Rosa hybrida* “Radrazz” rose bushes were grown in Angers, France, in three experiments. Experiment 1 took place in spring 2007 (2007-Sp), experiment 2 in summer 2007 (2007-Su), and experiment 3 in spring 2010, with a low (2010-LD) or high (2010-HD) plant density. Single-node cuttings bearing a five- or seven-leaflet leaf were harvested from the medial part of mother plant stems and grown for 4–5 weeks in humid conditions until rooting was achieved. Well rooted cuttings were planted in individual pots containing a mixture of neutral peat, coconut fibers and perlite. Plants were transferred to a greenhouse before bud break and grown, with a border row, at a density of 23 plants m^−2^ in experiments 1 and 2, and at a density of 21 or 100 plants m^−2^ in experiment 3. The bud from the cutting produced the primary axis. After floral bud and last leaf appearance, a variable number of lateral buds burst and developed in secondary shoots. These shoots were let to grow but not measured. Plants were subirrigated, with tensiometer monitoring to ensure an absence of water stress. Mineral nutrition was provided by fertigation (5.0 mM KNO_3_, 2.0 mM Ca(NO_3_)_2_, 2.0 mM NH_4_NO_3_, 2.0 mM KH_2_PO_4_, 2.0 mM MgSO_4_, 0.25 mM NaOH; trace elements (Kanieltra 6-Fe,0.1 ml.l^−1^, Hydro Azote, Nanterre, France); pH 5.6; EC 1.77 mS.cm^−1^). Air temperature was measured above the canopy, in a ventilated shelter, with a platinum sensor. Leaf temperature was measured in experiment 3 on the abaxial surface of leaves, at about the height of the apex, with copper-constantan thermocouples. Photosynthetically active radiation (PAR) was continuously measured above each canopy, with a line quantum sensor (LI-191 LI-COR, Lincoln, Neb. USA). It was also measured with line quantum sensors at both the base and the top of the canopy, at various locations, at solar noon, under a cloudy sky, on three dates during primary shoot expansion in experiment 3, for the calculation of intercepted PAR. The climatic conditions prevailing during the period of primary shoot expansion are presented in Table 1.

**Table 1:** **Prevailing climatic conditions and PAR interception during shoot expansion, at high and low plant density, in each experiment**.

**Experiment**	**2007 spring**	**2007 summer**	**2010 spring**		
				**High plant density**	**Low plant density**
Incident PAR (mol.m^−2^.day^−1^)	8.0 ± 1.6	11.7 ± 1.1		7.7 ± 1.1	
			Days after bud break		
Intercepted PAR (%)			15	72.3 ± 3.1	8.5 ± 0.2
			30	93.9 ± 1.0	37.8 ± 2.6
			36	95.0 ± 0.1	47.0 ± 2.1
Air temperature (daily mean, °C)	20.2 ± 1.9	21.7 ± 1.3		20.2 ± 0.9	
Leaf temperature (daily mean, °C)				22.3 ± 1.6	22.8 ± 1.4
Humidity (%)	57.1 ± 6.5	75.6 ± 4.5		56.1 ± 7.7	

Each value is the mean ± standard deviation.

### Plant measurements

#### Destructive measurements of leaf dimensions

Groups of 62, 17, and 18 plants were selected at random from the 2007-Sp, 2010-LD, and 2010-HD crops, respectively, and destructively sampled at various times (5–11 times, according to the crop), beginning when the basal leaves were expanding and ending when all the leaves were fully expanded. We determined the number of leaflets per leaf for all leaves (197, 201, 199 leaves for 2007-Sp, 2010-LD, and 2010-HD, respectively), the length and width of all leaflets and leaf length (measured with a ruler in 2007-Sp and by image analysis (ImageJ 1.43m, Wayne Rasband National Institute of Health, USA) in 2010-LD and 2010-HD). For 2010-LD and 2010-HD, we also determined the area of each leaflet by image analysis. For lateral leaflets, there was no significant difference between opposite leaflets for length, width or area, thus, for data analysis we used the mean value of length, width or area of each pair of opposite leaflets of an individual leaf.

#### Time-course measurements of visible leaf number, internode and terminal leaflet lengths

We randomly selected 33, 28, and 22 plants from the 2007-Su, 2010-LD, and 2010-HD crops, respectively. These plants were not those used for destructive measurements. The time of bud break of the cutting (referred to simply as bud break hereafter), marking the start of primary shoot development, was noted for each plant. Five times per week for 2007-Su and six times per week for 2010-LD and 2010-HD, we counted the number of visible leaves on each plant, including the scaly and stipular leaves located at the base of the shoot. A leaf was counted as visible as soon as its tip emerged. On primary shoots, we measured the length of the terminal leaflet of each leaf and the length of each internode with a ruler, from the first day on which the organ was fully visible until the end of organ expansion. A terminal leaflet was fully visible when its insertion on the rachis was visible, and an internode was fully visible when the node at its base was visible. Phytomer position of the organ was specified. The total number *N_p_* of phytomers, including the peduncle, of the primary shoot of the plant *p* was determined at the end of shoot expansion.

### Data analysis

#### Convention for phytomer position

For data analysis, organ position along the shoot was expressed in terms of the relative rank of the phytomer (*i*), such that the most basal or apical phytomers could be compared regardless of the number of phytomers per shoot. Relative rank was calculated as:

(1) $$i=(r-1)/(N_{p}-1)$$

where *r* is the absolute rank numbered from the base (*r* = 1) to the top (*r* = *N_p_*) of the shoot. The relative rank of the peduncle is therefore 1. For the figures, the rounded phytomer relative rank, the relative rank of the phytomer rounded to the nearest tenth (0.1), was used for the sake of clarity. A phytomer consists of an internode, the leaf at the top of the internode and its axillary bud.

#### Time of leaf appearance

We calculated thermal time from air temperature above the crop, using a base temperature of 2.1°C. This base temperature was determined for *Rosa hybrida* “Radrazz” in a previous experiment (Guerin, pers. com.) according to Yang et al. (1995). All variables defining the time of an event are expressed in degree days (°Cd) since cutting bud break.

**Figure 1:**
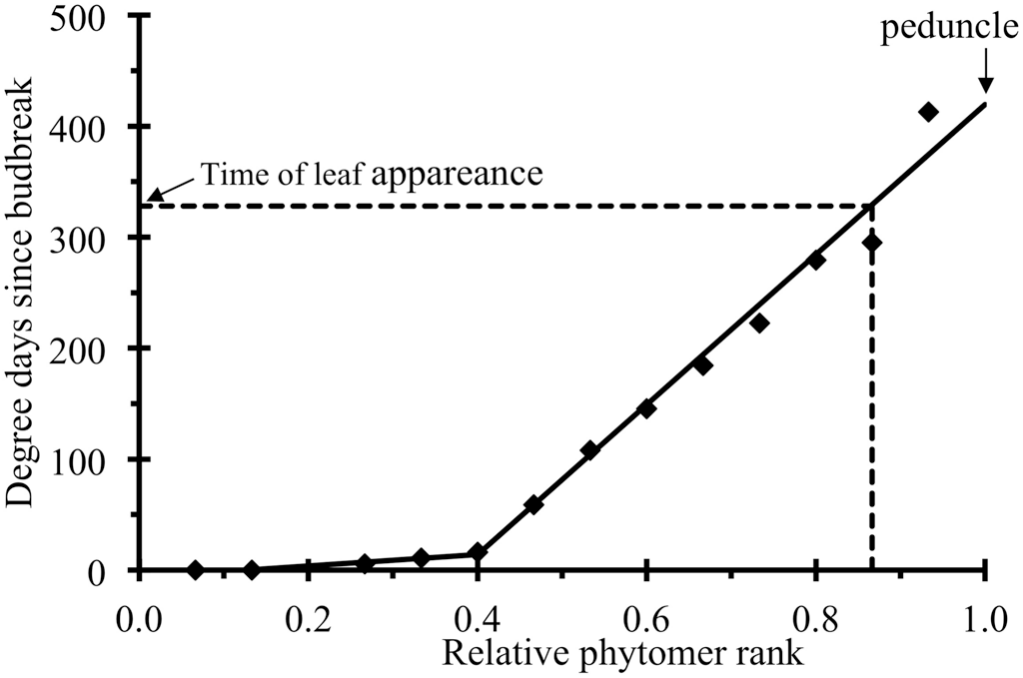
**Time (in degree days since budbreak) at which a leaf was observed for the first time (symbols) plotted against relative phytomer rank for an individual plant.** The lines correspond to the fitting of a piecewise linear function, as in Equation (2), to the experimental data.

The thermal time of leaf appearance *t^a^_p_* (*i*) for relative rank *i* of plant *p* could not be directly assessed by measurement, because the times at which the measurements were made did not correspond exactly to the time of leaf appearance. Thus, time *t^a^_p_* (*i*) was estimated from the parameters of a piecewise linear function fitted to the time of the first observation of each leaf (with or without leaflets) of plant *p* (Figure 1):

(2) $$t_{p}^{a}(i)=\{\begin{array}{ll} \alpha_{p}+\beta_{p}i & \text{if }i\leq c_{p}\\ \alpha_{p}+\beta_{p}i_{p}+\gamma_{p}(i-c_{p}) & \text{if }c_{p}<i\leq 1 \end{array}$$

where *c_p_* is the relative rank at which the two lines intersect, defining the transition between a phase of rapid leaf appearance and a phase of slow appearance, α_*p*_ is the thermal time at which the first leaf appeared, β_*p*_ and γ_*p*_ the phyllochrons (°Cd) for the leaves of relative ranks below and above *c_p_*, respectively. Peduncles do not bear leaves, but a date of “virtual leaf” appearance was calculated by linear extrapolation for the peduncle (*i* = 1).

#### Fitting of organ expansion

Each organ on the shoot was characterized by its kind (*k*, with *k* = *lea* for terminal leaflets and *k* = *int* for internodes) and relative rank *i*. If the final organ length exceeded 12 mm, the time course of length *L_p, k_* (*t, i*), expressed in mm, was fitted with a logistic function of the thermal time (*t*) since bud break of plant *p*:

(3) $$L_{p,\;k}(i,\;t)=\frac{L_{p,\;k}^{m}(i)}{1+\exp\left(4\frac{v_{p,\;k}^{m}(i)}{L_{p,\;k}^{m}(i)}\left(t_{p,\;k}^{0}(i)-t\right)\right)}$$

where *L^m^_p, k_* (*i*) is the maximal length of the organ, *t*^0^*_p, k_* (*i*) is thermal time at the inflexion point, referred to as time at mid-expansion hereafter, and *v^m^_p, k_* (*i*) is the expansion rate (mm °Cd^−1^) at the inflexion point (maximal expansion rate). The ratio *w^m^_p, k_* (*i*) = *v^m^_p, k_* (*i*)/*L^m^_p, k_* (*i*) is the maximal expansion rate when organ size is normalized, *i.e*., the maximal expansion rate of the ratio *L_p, k_* (*i, t*)/*L^m^_p, k_* (*i*). If final organ length was lower than 12 mm, which includes the internodes at the very base of the shoot for most plants and the internode just below the peduncle for a few plants, the thermal time course of organ length was not fitted with any function, because of a lack of accuracy, and the organ was discarded from the analyses using the fitting data.

For each individual organ whose final length exceeded 12 mm, we used the values of the parameters of the logistic function to calculate the duration of expansion, defined as the time required for the organ to expand from 10 to 90% of its final length. Duration of expansion of an organ is inversely proportional to the value of *w^m^_p, k_* (*i*) for this organ.

#### Modeling time of the inflexion point of the expansion function

The time *t*^0^*_p, k_* (*i*) of the inflexion point of the time course function of the organ *k* (*lea* for terminal leaflet, *int* for internode) at relative rank *i* of plant *p* was estimated from observed data as described in Fitting of Organ Expansion. We also aimed to predict the time of the inflexion point *t*^0^*_p, k_* (*i*) from the time of leaf appearance *t^a^_p_* (*i*), therefore *t*^0^*_p, k_* (*i*) was calculated as polynomials of the time of leaf appearance *t^a^_p_* (*i*) of the corresponding phytomer:

(4.1) $$t_{p,\;lea}^{0}(i)=\delta_{lea,\;0}+\delta_{lea,\;1}\;t_{p}^{a}(i)+\delta_{lea,\;2}\;t_{p}^{a}{\left(i\right)}^{2}$$

(4.2) $$t_{p,\text{ int}}^{0}(i)=\{\begin{array}{ll} \delta_{\text{int},\;0}+\delta_{\text{int},\;1}t_{p}^{a}(i) & \text{if }i<1\\ \delta_{ped,\;0}+\delta_{ped,\;1}t_{p}^{a}(i) & \text{if }i=1 \end{array}$$

The reasons for using such polynomials are given in the results section.

### Model description

#### Overview

The data analysis was used to develop a model reproducing the organ expansion kinetics of individual plants from a real crop of rose bush primary shoots, from input data. Organ length was simulated as a logistic function of thermal time since bud beak. In the reference scenario (S_0_), the input variables were chosen so as to capture variability in both final length and the time of organ expansion. Two other scenarios (S_1_ and S_2_), differing in the number of input variables, were compared with the reference scenario. In these three scenarios, the input variables selected were both easy to measure and non-destructive.

#### Reference scenario S_0_

***Scenario inputs.*** The input variables for each plant *p* are:

– (I1) The number *N_p_* of phytomers of *p* from which we derived the set of the relative ranks 0, 1/(*N_p_* − 1), …, (*N_p_* − 2)/(*N_p_* − 1), 1, using Equation (1).
– (I2) The time *t*^*a*, obs^_*p*_ (*i*) when the leaf of relative rank *i* (*i* = 0, …, (*N_p_* − 2)/(*N_p_* − 1)) of *p* was observed for the first time.
– (I3) The final length *L*^*m*, obs^_*p, lea*_ (*i*) of the terminal leaflet of relative rank *i* (*i* = 0, …, (*N_p_* − 2)/(*N_p_* − 1)) of *p*.
– (I4) The final length *L*^*m*, obs^_*p*, int_ (*i*) of the internode of relative rank *i* (*i* = 0, …, 1) of *p*.

***Scenario outputs.*** The outputs of the model consist of predicted functions. For each plant *p* of *N_p_* phytomers, the predicted length of its organ *k* (*k* = *lea* for terminal leaflet and *k* = *int* for internode) at relative rank *i*, expressed in thermal time *t* since bud break of *p* is

(5) $$L_{p,\;k}^{\text{pred}}(i,\;t)=\frac{L_{p,\;k}^{m,\text{ obs}}(i)}{1+\exp\left(4w_{k}^{m}(i)\;\left(t_{p,\;k}^{0,\text{ pred}}(i)-t\right)\right)}$$

where the parameters *t*^0, pred^_*p, k*_ (*i*) and *w^m^_k_* (*i*) are specified in the next paragraph.

***Parameter computations.*** From the observed times *t*^*a*, obs^_*p*_ (*i*), we estimated the parameters α_*p*_, β_*p*_, γ_*p*_, and *c_p_* of Equation (2), then we deduced the adjusted times (*t* ^*a*, adj^_*p*_ (*i*)) of leaf appearance. These appearance times were used to compute the predicted times *t*^0, pred^_*p, k*_ (*i*) corresponding to the inflexion points, using Equations (4.1) and (4.2).

First, we estimated the parameters $w_{p,\;k}^{m}(i)=\frac{v_{p,\;k}^{m}(i)}{L_{p,\;k}^{m}(i)}$ using the fitting procedure introduced in the paragraph Fitting of Organ Expansion for each organ kind, relative rank, and plant. Then, using these estimations for all plants, we estimated *w^m^_k_* (*i*) for each relative rank *i* with the LOESS method of SAS. In the model, the estimated value of *w^m^_k_* (*i*) thus depends on kind of organ and its relative rank, but not on the individual plant.

#### Scenario 1

***Scenario input.*** The time of leaf appearance is the most demanding of the input variables to measure, because it requires a series of observations to be taken at particular times, with little flexibility in timing possible. We therefore, decided to eliminate this observation from scenario S_1_. So the input variables of each plant *p* are (I1), (I3), and (I4).

***Scenario outputs.*** The output functions of the scenario S_1_

(6) $$L_{p,\;k}^{\text{pred}}(i,\;t)=\frac{L_{p,\;k}^{m,\text{ obs}}(i)}{1+\exp\left(4w_{k}^{m}(i)\left(t_{k}^{0}(i)-t\right)\right)}$$

are similar to the outputs of the scenario S_0_ Equation (5), except that the thermal time at mid-expansion is independent of the plant and depends only on relative rank. The parameter *w^m^_k_* (*i*) is specified in the paragraph Reference Scenario S_0_ Parameter Computations and the parameter *t*^0^_*k*_ (*i*) is specified in the next paragraph.

***Parameter computations.*** In S_1_, the value *t*^0^_*k*_ (*i*) was estimated by relating the values *t*^0^_*p, k*_ (*i*), estimated with the fitting procedure introduced in the paragraph Fitting of Organ Expansion, with relative rank *i* by the LOESS method of SAS.

#### Scenario 2

***Scenario input.*** Scenario S_2_ simulates the kinetics of expansion of plants differing only in phytomer number. The comparison of S_0_ with S_2_ was designed to estimate the gain in accuracy with the use of a model using *n* different individual plants to simulate expansion (S_0_) rather than a model reproducing *n* mean plants differing only in phytomer number (S_2_). In S_2_, the only input variable is the number of phytomers per primary shoot (I1), which is used to calculate relative ranks.

***Scenario output.*** The output of the scenario S_2_ is independent of the plant in the sense that the organ lengths of plants with the same number of phytomers are similar:

(7) $$L_{k}^{\text{pred}}(i,\;t)=\frac{L_{k}^{m}(i)}{1+\exp\left(4w_{k}^{m}(i)\left(t_{k}^{0}(i)-t\right)\right)}$$

where the parameters *w^m^_k_* (*i*) and *t*^0^_*k*_ (*i*) are previously specified and the parameter *L^m^_k_* (*i*) is specified in the next paragraph.

***Parameter computations.*** The final organ length *L^m^_k_* (*i*) was estimated by relating the measured values of *L^m^_p, k_* (*i*) to *i*, with the LOESS method of SAS.

### Model evaluation

The three scenarios were evaluated by the leave-one-out cross-validation method (Linhart and Zucchini, 1986).

For each plant *p* of the original data set consisting of *n* plants:

– We removed plant *p* and estimated the model parameters from the *n* − 1 remaining plants.
– We computed the expansion curves for each terminal leaflet and each internode of plant *p*, using the scenario output, and calculated the mean square error of prediction:
  (8) $$MSEP_{p,\;k}(i)=\frac{\sum_{t=1}^{n_{p,\;k}(i)}{\left(L_{p,\;k}^{\text{obs}}(i,\;t)-L_{p,\;k}^{\text{pred}}(i,\;t)\right)}^{2}}{n_{p,\;k}(i)}$$

where *n_p, k_* (*i*) is the number of observations for an organ of kind *k* of plant *p* of relative rank *i*, *L*^obs^_*p, k*_ (*t, i*) is the observed organ length at time *t* and *L*^pred^_*p, k*_ (*i, t*) (or *L*^pred^_*k*_ (*i, t*) for S_2_) is the predicted organ length at time *t* computed from one of the functions given in Equations (5–7). For *MSEP_p, k_* (*i*) calculations, we retained only four values for the plateau, defined as the time at which organ length exceeded 0.97 times the final length, to avoid giving too much weight to the plateau.

Mean *MSEP_p, k_* (*i*) was then calculated for all plants, both per rounded relative rank and for all phytomer relative ranks pooled together. The square root of MSEP (RMSEP) was then calculated.

### Statistical analyses

Principal component analyses were performed with SPAD software (V7.4, Coheris, Suresnes, France). All other statistical analyses were performed with SAS software (V 9.3, SAS Institute). For the fitting of logistic functions Equation (3) and of piecewise linear functions Equation (2), we used the NLIN procedure. For the establishment of relationships between relative rank *i* on the one hand and *w^m^_k_* (*i*), *t*^0^_*k*_ (*i*), or *L^m^_k_* (*i*) on the other, we used non-parametric methods and the LOESS procedure.

## Results

We will first present the variability present in the datasets used for expansion studies. We will then propose a simplified representation of rose compound leaves for studies of the kinetics of expansion, and describe the principal features of organ expansion kinetics: global pattern, origin and amplitude of the variability and how this variability can be captured through relationships. Finally, we will present a model based on this analysis that simulates leaflet and internode expansion kinetics from non-destructive input data.

### Architectural variables were highly variable both between phytomer positions and between plants

High levels of variation were observed in all three crops used for time-course measurements. Primary shoots comprised between 10 and 16 phytomers on the plants used for time-course measurements (Table 2). The rate of leaf appearance was highly variable, with the phyllochron during the phase of slow leaf appearance varying from 20.6 to 53.4°Cd between plants within a crop, resulting in considerable variation of the dates of leaf appearance at equivalent phytomer positions (e.g., at rank 10, leaf appearance on the first plant occurred 172°Cd before that on the last plant within a crop). Final lengths were between 6.5 and 87.5 mm for terminal leaflets and between 1.0 and 63.5 mm for internodes, considering all ranks together. At a given phytomer position, final lengths also varied considerably between plants. For example, at a rounded relative rank of 0.8, the range of final lengths was 58.0–86.0 mm for terminal leaflets and 9.0–49.5 mm for internodes. Differences in mean values between crops, for phytomer numbers, timing of leaf appearance and final lengths, were significant.

**Table 2:** **Mean values and variability of architectural variables for the three crops used for the expansion study**.

	**Phytomers per shoot**	**Phyllochron during the phase of slow leaf appearance (°Cd)**	**Time of leaf appearance at rank 10 (°Cd since bud break)**	**Final lengths (mm), all ranks together**		**Final lengths (mm) at relative rank 0.8**	
				**Terminal leaflet**	**Internode**	**Terminal leaflet**	**Internode**
**2007-Su**							
Mean ± std	12.0 ± 0.9 c^a^	35.0 ± 7.8 b	198.0 ± 28.3 a	60.6 ± 12.2 ab	21.0 ± 12.8 b	71.5 ± 5.4 b	27.8 ± 6.7 b
Min-max	10–13	21.2–50.7	152.3–264.3	18.0–83.0	1.0–62.0	58.0–80.0	9.0–40.0
**2010-LD**							
Mean ± std	14.4 ± 1.0 a	35.5 ± 5.9 b	127.1 ± 24.4 c	62.4 ± 16.7 a	23.9 ± 14.1 a	77.5 ± 5.1 a	36.0 ± 5.5 a
Min-max	13–16	24.5–46.8	79.0–173.4	6.5–87.5	3.5–61.5	63.0–86.0	22.5–48.0
**2010-HD**							
Mean ± std	13.5 ± 1.4 b	39.7 ± 7.9 a	159.5 ± 47.6 b	58.2 ± 16.0 b	24.5 ± 16.0 a	71.0 ± 6.2 b	34.9 ± 7.7 a
Min-max	10–16	20.6–53.4	87.0–258.6	6.5–82.0	3.0–63.5	59.0–82.0	16.0–49.5

a Mean values with the same letters do not differ significantly between crops at P < 0.05, in One-Way ANOVA followed by LSD tests. Variability was assessed by determining both the standard deviation and the minimal and maximal values within each crop.

### Terminal leaflet length can be used to simulate the dimensions of all leaflets within a leaf

Rose leaves have generally odd numbers of leaflets, with between one and nine leaflets per leaf, except at the base of the shoot, where leaves are reduced to scales or stipules. Principal component analysis (PCA) was performed on the lengths, widths and square roots of the areas of all individual leaflets of both growing and mature leaves collected from the 2007-Sp, 2010-LD, and 2010-HD crops. The percentage of the variance explained by the first principal component was 97, 79, 79, and 80% for 1-, 3-, 5-, and 7-leaflet leaves, respectively (no nine-leaflet leaf sampled in these experiments). These very high values for a single component indicate that all the dimensions (lengths, widths and square roots of areas) of all the leaflets were correlated within a given leaf, for both growing and mature leaves. Consequently, we were able to simplify the study of leaf expansion by focusing on a single dimension. We chose to study terminal leaflet length, because all leaves composed of leaflets have a terminal leaflet and this dimension is easily measured earlier in expansion than the other possible dimensions.

Terminal leaflet length (*L*) can be used to calculate whole leaf area (*A*) at any growth stage, given the number of leaflets of the leaf (*N_L_*), from the following equation:

(9) $$A=0.287\;L^{2}\;N_{L}^{0.746}\;(n=201,\;R^{2}=0.93)$$

With a multiplicative model for terminal leaflet length and leaflet number per leaf, the residual distribution did not depend on leaflet number per leaf as with a linear model. The two parameter values (0.287 and 0.746) were adjusted so as to maximize *R*^2^.

### Expansion follows a sigmoidal pattern that can be fitted by logistic function

**Figure 2:**
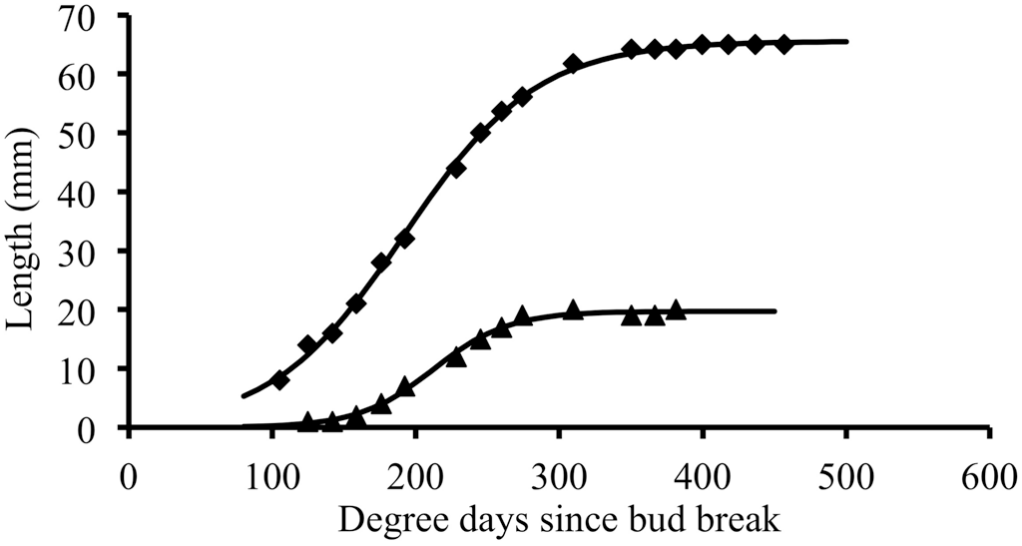
**Thermal time course of terminal leaflet (diamond) or internode (triangle) length for an individual organ.** Symbols represent experimental data and the lines correspond to the symmetric logistic function fitted to the experimental points.

In individual organs, changes in terminal leaflet or internode length as a function of thermal time followed a sigmoidal pattern (Figure 2). This pattern of change was well fitted by a logistic function (Table 3): *R*^2^ close to 1 and RMSEP around 1 mm. For all phytomers, the period of internode expansion was included within the expansion period of the terminal leaflet of the same phytomer (Figure 2). Terminal leaflets reached 10% of their final size a mean of 31°Cd before the corresponding internodes reached 10% of their final size, and the leaflets reached 90% of their final size a mean of 35°Cd after the internodes.

**Table 3:** **Goodness-of-fit of logistic functions to expansion curves of individual terminal leaflets and internodes: number of fitted curves, mean values of *R*^2^, root mean square error (RMSE), and mean number of observations per curve**.

	**Terminal leaflets**	**Internodes**
Number of organs for which expansion curves were fitted	622	711
*R*^2^	0.99	0.98
RMSE (mm)	1.2	0.81
Number of observations per curve	19	16

### Differences in expansion kinetics are largely due to differences in the time at which expansion occurs and to maximal expansion rate

#### Between plants at a given phytomer position

**Figure 3:**
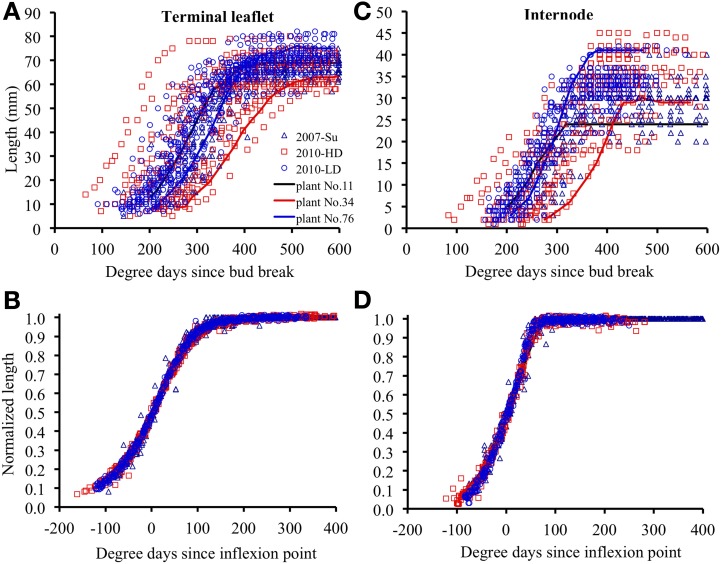
**Elongation of individual organs positioned at relative rounded rank 0.7, as a function of thermal time since bud break, for terminal leaflets (A) or internodes (C). (B,D)** show the same data for normalized length (length at any time divided by final length) and for thermal time counted since the inflexion point of the expansion curve of each individual organ. The crops 2007-Su, 2010-LD, and 2010 HD are represented by different symbols and colors. Each point corresponds to the measurement of an individual organ. Examples of individual curves are given for three plants (plants 11, 34, and 76).

Within each relative rank, the variability of terminal leaflet expansion kinetics was high, as shown for relative rank 0.7 in Figure 3A. This variability resulted from high variability of both size and the time at which expansion occurred (265°Cd separating the times at mid- expansion of the first and last plants). All individual observations were almost superimposable if the length of each organ at a given time was normalized by dividing by its final length, and if the time was expressed as time since mid-expansion of individual organ (*t*^0^_*p, k*_ (*i*); Figure 3B). Thus, the differences in organ size between plants essentially resulted from differences in maximal expansion rate, rather than differences in expansion duration. This was true for all relative ranks, for both terminal leaflets and internodes (Figures 3C,D).

#### Between phytomer positions

**Figure 4:**
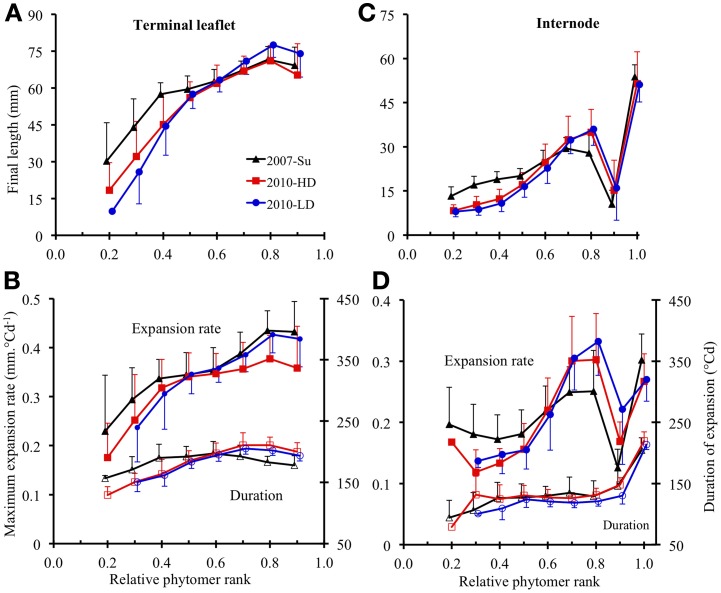
**Final length (A,C), maximum expansion rate (B,D, closed symbols) and expansion duration (B,D, open symbols) plotted against rounded relative phytomer rank for terminal leaflets (A,B) and internodes (C,D).** The 2007-Su, 2010-LD, and 2010-HD crops are represented by different symbols and colors: black triangles for 2007-Su, blue circles for 2010-LD and red squares for 2010-HD. Error bars represent the standard deviation, with 33, 28, and 22 plants in the 2007-Su, 2010-LD, and 2010-HD crops, respectively.

For terminal leaflets, the variations of final length and maximal expansion rate with phytomer position followed the same tendencies for all three crops: a strong increase in the basal part of the shoot (up to relative ranks 0.4–0.5), then a more moderate increase up to relative rank 0.8, followed by a slight decrease or stability (Figures 4A,B). Expansion duration increased moderately from the base to relative rank 0.4 (2007-Su) or 0.7 (2010-LD and 2010-HD) and then stabilized and decreased for upper ranks (Figure 4B).

For internodes, final length (Figure 4C) increased up to relative ranks 0.8 then strongly decreased for relative rank 0.9 and increased strongly for relative rank 1.0 (peduncle). The variations of maximal expansion rate followed an essentially similar pattern to final length, except at the base of the axis (Figure 4D). By contrast, variations of expansion duration along the shoot (Figure 4D) were characterized by stability from relative ranks 0.3–0.9, with a lower duration at relative rank 0.2 and a much greater duration (×1.7) for the peduncle.

For both terminal leaflets and internodes, absolute values of final length, maximal expansion rate and expansion duration differed between the crops; in addition, the ranking of the three crops for these variables differed between different zones of the shoot. However, for all crops, the amplitude of variation between phytomer relative ranks was much higher for final length and maximal expansion rate than for expansion duration.

Within each crop, plants with a high leaflet or internode final length at a particular phytomer position did not necessarily also have a greater length at the next phytomer (not shown); the same was true for low leaflet or internode final lengths.

### Time of leaf appearance can be used to estimate time at mid-expansion

**Figure 5:**
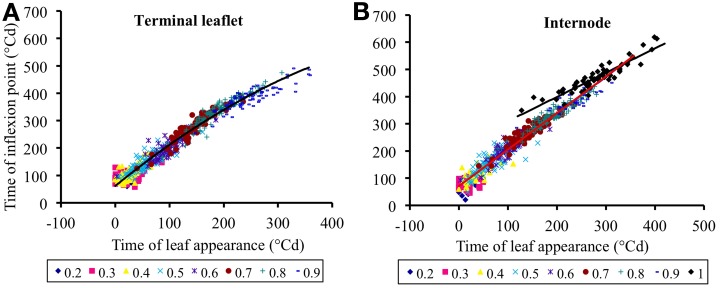
**Time at the inflexion point of the expansion curve for leaflets (A) or internodes (B), plotted against the time of leaf appearance.** All times are expressed in degree days since bud break. Each point corresponds to an individual organ. Data corresponding to the same rounded relative phytomer are represented by the same symbol, without distinction between the three crops (2007-Su, 2010-LD, and 2010-HD). The lines correspond to Equation (4), parameter values are given in Table 5.

Figure 5 shows the mid-expansion times for individual leaflets (*n* = 629) and internodes (*n* = 584) plotted against time of leaf appearance. These relationships integrate both the rate at which expansion occurs on successive phytomers and the duration of expansion, because the predicted variable is the time at the middle and not at the beginning of expansion. For terminal leaflets, the relationship between time at mid-expansion and time of leaf appearance gradually curves, because expansion duration changes gradually with phytomer relative rank (Figure 4B). We therefore decided to use second-order polynomials to adjust the relationship for terminal leaflets. For the internodes, expansion duration was almost stable for vegetative internodes and much longer for peduncles (Figure 4D). We therefore, used first-order polynomials to adjust the relationship between time at mid-expansion and time of leaf appearance, using different parameters for vegetative internodes and peduncles. Estimates for the parameters of the relationships defined in the Equations (4.1) and (4.2) are given in Table 4.

**Table 4:** **Values of the model parameters used in the reference scenario (S_0_) other than those obtained by the LOESS method (see Table 5)**.

**Parameter**	**Equation**	**Value**
δ_*lea*,0_ (°Cd)	4.1	61.5
δ_*lea*,1_	4.1	1.62
δ_*lea*,2_ (°Cd^−1^)	4.1	−0.00113
δ_*int*,0_ (°Cd)	4.2	70.6
δ_*int*,1_	4.2	1.34
δ_*ped*,0_ (°Cd)	4.3	220
δ_*ped*,1_	4.3	0.893

The intercepts δ_*lea*,0_ and δ_*int*,0_ correspond, for phytomers whose leaf appears at bud break, to the time lag between leaf appearance and organ (leaflet or internode, respectively) maximal expansion rate. The other parameters of Equations (4.1) and (4.2) have no direct biological meaning. The adjusted determination coefficients were *R*^2^_adj_ = 0.97 (*n* = 627) for terminal leaflets, *R*^2^_adj_ = 0.96 (*n* = 525) for vegetative internodes and *R*^2^_adj_ = 0.86 (*n* = 59) for peduncles. These high *R*^2^ indicate that time of leaf appearance can be used to estimate time at mid-expansion for individual leaflets and internodes, because these relationships capture the variability between crops, between individual plants and between phytomer positions.

**Table 5:** **Model parameters: values of *w^m^_k_* (*i*), *t*^0^_*k*_ (*i*), and *L^m^_k_* (*i*) estimated from the experimental data by the LOESS method**.

**Relative rank**	**Terminal leaflets**			**Internodes**		
	***w^m^_lea_* (*i*) × 10^3^**	***t*^0^_*lea*_ (*i*)**	***L^m^_lea_* (*i*)**	***w^m^_int_* (*i*) × 10^3^**	***t*^0^_*int*_ (*i*)**	***L^m^_int_* (*i*)**
	**(°Cd)**	**(°Cd)**	**(mm)**	**(°Cd)**	**(°Cd)**	**(mm)**
0.0						5.9
0.05						7.0
0.1					23.7	9.0
0.15	7.39	74.7	19.6		38.8	10.4
0.2	7.16	82.4	27.1	11.72	54.9	11.7
0.25	6.94	89.7	34.3	10.99	70.5	12.5
0.3	6.72	97.1	41.5	10.35	86.1	13.0
0.35	6.53	105.0	46.9	9.76	100.4	13.3
0.4	6.31	116.6	52.5	9.27	118.8	14.6
0.45	6.11	132.9	55.3	8.88	138.8	16.1
0.5	5.93	154.3	57.8	8.66	162.6	17.9
0.55	5.78	180.7	60.1	8.76	189.4	20.4
0.6	5.66	211.6	62.7	8.86	217.7	23.4
0.65	5.56	250.3	65.9	8.95	250.3	28.2
0.7	5.53	281.9	68.4	8.97	277.0	31.7
0.75	5.54	313.6	71.1	8.94	306.1	33.6
0.8	5.60	340.2	73.1	8.77	334.8	32.4
0.85	5.70	362.6	73.4	8.43	363.9	26.1
0.9	5.79	381.4	72.2	7.94	394.6	14.4
0.93	5.84	393.3	71.4	7.34	414.2	8.0
1.0				5.19	457.6	52.7

*w^m^_k_* (*i*) *is used in scenarios S_0_, S_1_ and S_2_; t*^0^_*k*_ (*i*) *is used in scenarios S_1_ and S_2_; L^m^_k_* (*i*) *is used in scenario S_2_.*

### A model of leaflet and internode expansion reproduces interplant variability

From the results presented above, we developed a model reproducing the terminal leaflet and internode expansion of a crop of rose bush primary shoots. The three scenarios of the model, differing in the number of input variables, were evaluated by cross-validation.

**Figure 6:**
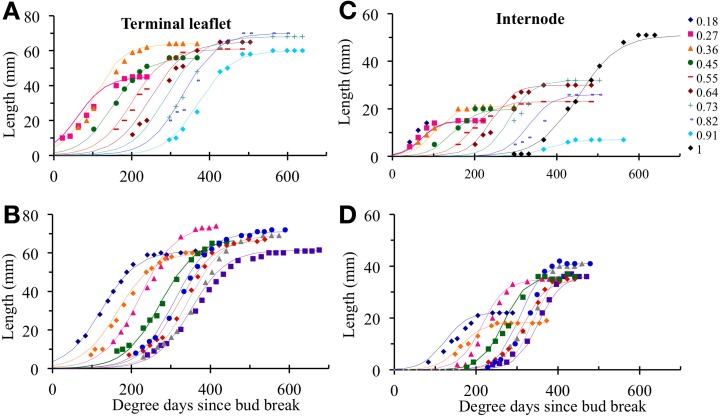
**Observed (symbols) and simulated (lines) organ lengths as a function of thermal time for the different relative ranks of an individual plant (A,C) or for different plants at the same relative rank (B,D).** Illustrations **(A,B)** show terminal leaflet lengths, **(C,D)** show internode lengths. As an example, an individual plant was chosen for illustrations **(A,C)**, and a rounded relative rank of 0.7 is used for illustrations **(B,D)**. Simulated values were obtained with the cross-validation method.

**Figure 7:**
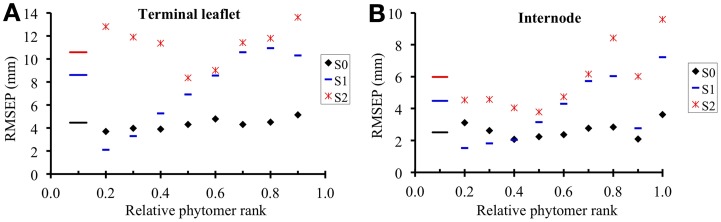
**Square root of the mean square error of prediction (RMSEP) for terminal leaflet (A) and internode (B) expansion, plotted against rounded relative phytomer rank.** The short horizontal lines show the value of RMSEP for all phytomer positions considered together. RMSEP was calculated throughout the expansion of each individual organ, according to the three scenarios of the model. In S_0_ (reference scenario) input variables account for the interplant variability in both organ final length and time of leaf appearance; in S_1_, input variables account for the interplant variability in organ final length; S_2_ simulates mean plants.

In the reference scenario (S_0_), input variables accounted for interplant variability in both organ final lengths and time of leaf appearance. The term *w^m^_k_* (*i*) depends only on phytomer position and does not vary between individual plants. This term represents the maximal expansion rate for a normalized expansion curve and is inversely proportional to expansion duration. The parameters of S_0_ are given in Table 4 and the values of *w^m^_k_* (*i*) estimated by the LOESS method are given in Table 5. S_0_ correctly reproduced the expansion kinetics of the different terminal leaflets and internodes of an individual plant (Figures 6A,C) and of different plants within a given crop (Figures 6B,D). The RMSEP for all ranks together was 4.5 mm for leaflets and 2.5 mm for internodes, corresponding to 7.3 and 10.2%, respectively, of organ final lengths. The accuracy of the model was similar for the different phytomer positions, for both leaflets (Figure 7A) and internodes (Figure 7B).

Scenario 1 was designed to assess model accuracy assuming that the time at which expansion occurred depended only on phytomer position, rather than being estimated, as in S_0_, from an input variable. The parameters of S_1_ are given in Table 5. In S_1_, the RMSEP for all ranks together was 8.6 mm for leaflets and 4.5 mm for internodes (14.2 and 18.2% of final lengths, respectively). The RMSEP calculated for each phytomer position increased from the base to the top of the shoot (Figure 7). This was due to an increase in variability between plants for mid-expansion time, from the base to the top of the shoot (Figure 5). The optimal scenario would thus involve estimating the time at mid-expansion from a value dependent only on relative rank for basal phytomer positions and from measurements of leaf appearance time for more central and apical positions. In practical terms, this would decrease measurement time with respect to S_0_, because the leaf-like structures at the base of the shoot are the most difficult to observe.

Scenario 2 simulates the kinetics of expansion of plants differing only in terms of phytomer number. The parameters of S_2_ are given in Table 5. For S_2_, RMSEP was calculated by plant, so the comparison of the RMSEP values for S_0_ and S_2_ provides an estimate of the gain in accuracy when a scenario accounting for interplant variability (S_0_) is used to simulate the expansion kinetics of a heterogeneous crop over a scenario reproducing *n* mean plants. In S_2_, RMSEP for all ranks together was 10.6 mm (17.4 %) for leaflets and 6.0 mm (24.2%) for internodes, these values being 2.4 times higher than those for S_0_. This loss of accuracy for S_2_ with respect to S_0_ concerned all phytomer positions (Figure 7). It should be borne in mind that the method used to calculate MSEP does not aim to assess the ability of S_2_ to simulate a mean plant, because simulated lengths were not compared with mean observed lengths.

## Discussion

The aim of this study was to assess the kinetics of expansion of rose bush primary shoot internodes and leaves and to propose an empirical model reproducing organ expansion kinetics from non-destructive, easy-to-measure variables. Bush roses are ornamental and are sold as individual plants. We therefore, paid particular attention to interplant variability in expansion kinetics and to the ability of the model to simulate this variability.

### Growing conditions and inter- and intra-crop variability

We studied expansion in three crops (2007-Su, 2010-LD, and 2010-HD) subjected to different growing conditions, contrasting in terms of incident PAR, humidity and plant density in particular, to ensure that we obtained robust results. Contrasted experimental conditions were used to find regularities in expansion kinetics, but not to establish quantitative relationships between architectural traits and environmental factors, such as density, light intensity or quality. This would require specific experiments. The large differences in PAR interception between densities in 2010 (Table 1) suggest that light quality in the canopies, including the red-far red ratio in particular, differs between plant densities. These differences in growing conditions resulted in significant differences between crops in terms of the mean values of architectural variables. Within each growing condition, the plants also displayed considerable variability. This level of interplant variability is typical of rose bush crops. This variability was observed although we selected cuttings to reduce heterogeneity due to topophysis (Bredmose et al., 2001) and differences in cutting leaf area (Costa and Heuvelink, 2003).

### At any time during expansion, the dimensions of the whole leaf can be reconstituted from terminal leaflet length

Our results show that it is possible to reconstitute the dimensions of the different leaflets of a rose leaf from a single dimension at any time during leaf expansion, given the number of leaflets per leaf. These results generalize those of Gao et al. (2012), who established that, for fully expanded leaves, total leaf area could be inferred from one leaf dimension and number of leaflets, within a genotype. The allometric relationships using terminal leaflet length as a predictor of either whole leaf area (presented here, section Terminal Leaflet Length can be Used to Simulate the Dimensions of all Leaflets Within a Leaf) or leaflet dimensions (Demotes-Mainard et al., 2009) should probably be established for each genotype. Indeed, only relationships based on the product of length by width have been found to be stable across a range of genotypes, both at the leaflet level (Rouphael et al., 2010) and the leaf level (Gao et al., 2012), because the shape of leaflets and their insertion position along the rachis (loose or close) vary between genotypes.

### Stability of organ expansion duration and variability of final organ size

Differences in organ final size between phytomer positions and between plants at a given position resulted mainly from differences in maximal expansion rate, rather than expansion duration. This is similar to the behavior of other species. For example, whereas final leaf and internode size widely varies between phytomers along the main shoot, organ expansion duration little varies in narrow-leafed lupin (Dracup and Kirby, 1993) and is stable in arabidopsis (Mündermann et al., 2005). At a given phytomer position, expansion duration is stable in thermal time between plants grown in contrasted growing conditions for sunflower leaves (Granier and Tardieu, 1998; Dosio et al., 2003), sorghum leaves (Lafarge and Tardieu, 2002) and internodes (Xue et al., 2012). In tall fescue and wheat, expansion duration is more stable if expressed in phyllochronic time (thermal time divided by the phyllochron) than if expressed in thermal time (Fournier et al., 2005). This stability in phyllochronic time has been interpreted as an emerging property of a self-regulated system, in which the appearance of an organ triggers changes in expansion (Fournier et al., 2005; Verdenal et al., 2008). Under this hypothesis, the link between phyllochron and duration results from a large proportion of expansion occurring when the leaf is still in the whorl generated by previous leaves.

In rose bush, as in many other species (for example lupin, Dracup and Kirby, 1993; wheat, Evers et al., 2005; cucumber, Kahlen, 2006; sorghum, Xue et al., 2012), there was a general gradient of final organ length with phytomer relative rank common to all three crops and plants. This gradient can be used to model a mean plant, as in scenario S_2_. However, this gradient varied between individual plants: a plant with a high (or low) leaflet or internode final length at a particular phytomer position did not systematically present a high (or low) length at the next phytomer. This was unexpected, at least for basal phytomer positions. Indeed, during their expansion basal phytomers depend on the cutting, both for nitrogen, which is provided by remobilization (Cabrera, 2003) and for photosynthesis (Costa and Heuvelink, 2003). In addition, on average eight phytomers closest to the base of the plant are preformed in the bud in the variety studied here (Girault et al., 2008), suggesting that they may be influenced similarly by the physiological state of the mother stem. We therefore, assume that the differences in leaflet or internode final size between plants may reflect differences in the phylloclimate perceived by the organs during their expansion. Indeed, final size is dependent, in several species, on phylloclimate (or climate) at the time of organ expansion (Granier and Tardieu, 1999, for sunflower leaves; Gautier et al., 2000, for white clover internodes, petioles and leaves; Andrieu et al., 2004, for maize leaves; Kahlen and Stützel, 2011, for cucumber internodes). Our model could be used to test this hypothesis, by accurately defining the timing of individual organ expansion.

### Modeling the expansion of individual primary shoots

We developed a model reproducing the kinetics of leaflet and internode expansion on rose bush primary shoots, based on non-destructive and easy-to-measure input variables. The comparison of the three scenarios showed that, to reproduce accurately the variability of expansion within a crop, it was necessary to measure the final lengths of terminal leaflets and internodes and to count the number of phytomers per shoot and of leaflets per leaf, all of which can be done after the final primary shoot has ceased to elongate. The timing of leaf appearance must also be established, at least for leaves in the middle and upper part of the shoot, as fixed values of time at mid-expansion can be used for the basal phytomers. These data are easy to acquire, but their acquisition is labor-intensive. The model based on these input variables satisfactorily simulated the diversity of individual plants. For ecophysiological studies, this model will make it possible to relate traits of interest measured on specific plants to features of expansion for the same plants, which is important for experimental crops with high levels of interplant variability.

In order to represent architectural development, our model of organ expansion can be easily integrated in a FSPM of rose bush using L-systems. The addition of a three-dimensional structure to the expansion model implies to model organs' shape and spatial position, as done for FSPMs of other species (e.g., Evers et al., 2005; Mündermann et al., 2005; Kahlen et al., 2008). This 3D model would provide essential information to study the environmental regulation of bud break, which strongly influences plant architecture and still remains a major research area (Domagalska and Leyser, 2011). Indeed, when coupled with a light model, the model will give information about light phylloclimate, which is a key factor controlling bud break through carbohydrate availability (Girault et al., 2010; Henry et al., 2011; Rabot et al., 2012), and light quality (Mor and Halevy, 1984; Girault et al., 2008). In rose bush, there is considerable interplant variability in the number and position of buds breaking along the primary shoot (for instance, in 2007-Su between 2 and 8 buds outgrew on primary shoots with the same number of axillary buds). It may therefore, be particularly relevant to be able to investigate the relationships between light phylloclimate and bud break at the level of individual plants.

The differences in organ expansion kinetics between plants within a relative rank resulted mainly from differences in maximal expansion rate, rather than differences in expansion duration. This had two related implications, which were used in the model. Firstly, variations in organ final size were sufficient to capture interplant variability in size at any time during expansion, within a relative rank, as in scenarios S_0_ and S_1_. Secondly, in the model, the term *w^m^_k_ (i)*, which is inversely proportional to expansion duration, can be predicted from a single value common to all plants within a relative rank with little loss of accuracy (all scenarios). In the model the term *w^m^_k_* (*i*) depends on relative rank. However, the variations of expansion duration between phytomer positions were moderate for terminal leaflets and for vegetative internodes. In scenario S_0_, if *w^m^_k_* (*i*) was replaced by only three values, one common to all terminal leaflets (*w^m^_lea_* = 5.96 × 10^−3°^Cd), one common to all vegetative internodes (*w^m^_int_* = 9.04 × 10^−3°^Cd) and one for peduncles (*w^m^_ped_* = 5.17 × 10^−3°^Cd), the accuracy of the model remained the same (RMSEP = 7.4 and 10.2% of final length, for terminal leaflets and internodes, respectively, data not shown) as with *w^m^_k_* (*i*). Thus, if the model has to be calibrated for a new genotype, this process could be simplified by estimating the model parameters from a limited number of phytomer positions.

The time of leaf appearance has been used in several architectural models for the prediction of ontogenic development (for example in wheat: Fournier et al., 2003; Evers et al., 2005; in cucumber: Kahlen, 2006; in ryegrass: Verdenal et al., 2008). In our model, we used this parameter in the reference scenario S_0_ to predict the mid-expansion time for both leaflets and internodes. This coordinates expansion between different phytomer positions for the same kind of organ, and between leaflets and internodes.

## Conclusion

This work provides insight into the kinetics of expansion at the level of individual organs that was lacking for rose bush. It is original in that it considers interplant variability, which has been little studied, in any plant species. This variability is important for rose bushes, because crops display high levels of interplant variability for architecture and the commercial product is the individual plant. On the basis of these results, we propose an empirical model that accurately reproduces, for individual plants, the expansion kinetics of primary shoots from non-destructive input variables. Even in its current state, this model already provides a useful tool for studying ecophysiological processes, such as bud break response to light phylloclimate, because a primary shoot constitutes an interesting model of the whole plant, with simple interactions between organs. With a view to studying rose bush architecture, these results can be used as a grid for analyzing the expansion of shoots resulting from branching, and for investigating genotype differences.

### Conflict of interest statement

The authors declare that the research was conducted in the absence of any commercial or financial relationships that could be construed as a potential conflict of interest.
